# Dengue virus neutralizing antibody: a review of targets, cross-reactivity, and antibody-dependent enhancement

**DOI:** 10.3389/fimmu.2023.1200195

**Published:** 2023-06-02

**Authors:** Animesh Sarker, Nidhi Dhama, Rinkoo Devi Gupta

**Affiliations:** Faculty of Life Sciences and Biotechnology, South Asian University, New Delhi, India

**Keywords:** dengue virus, neutralizing antibodies, cross-reactivity, antibody engineering, antibody-dependent enhancement, subunit vaccine

## Abstract

Dengue is the most common viral infection spread by mosquitoes, prevalent in tropical countries. The acute dengue virus (DENV) infection is a benign and primarily febrile illness. However, secondary infection with alternative serotypes can worsen the condition, leading to severe and potentially fatal dengue. The antibody raised by the vaccine or the primary infections are frequently cross-reactive; however, weakly neutralizing, and during subsequent infection, they may increase the odds of antibody-dependent enhancement (ADE). Despite that, many neutralizing antibodies have been identified against the DENV, which are thought to be useful in reducing dengue severity. Indeed, an antibody must be free from ADE for therapeutic application, as it is pretty common in dengue infection and escalates disease severity. Therefore, this review has described the critical characteristics of DENV and the potential immune targets in general. The primary emphasis is given to the envelope protein of DENV, where potential epitopes targeted for generating serotype-specific and cross-reactive antibodies have critically been described. In addition, a novel class of highly neutralizing antibodies targeted to the quaternary structure, similar to viral particles, has also been described. Lastly, we have discussed different aspects of the pathogenesis and ADE, which would provide significant insights into developing safe and effective antibody therapeutics and equivalent protein subunit vaccines.

## 1 Introduction

Dengue epidemiology has grown dramatically with dengue fever, now endemic in 128 tropical and subtropical nations, where more than half of the world’s population lives. The data analysis from Global Burden of Diseases, Injuries, and Risk Factors Study (GBD) 2017 revealed 107.6% increase in disability-adjusted life years (DALYs) since 1990. Dengue incident cases and age-standardized incidence rates (ASR) increased from 30.7 million and 557.15 per 100,000 people in 1990 to 56.9 million and 740.4 per 100,000 in 2019, respectively, as per the GBD 2019 data study. The three largest endemic regions with the greatest dengue incidence in 2019 are Oceania, South Asia, and Southeast Asia (1). Dengue has spread throughout the Pacific and the Americas as a result of international trade and tourism (2). Due to worldwide travel and warming, the prevalence of dengue is rapidly rising (3).

Dengue virus (DENV) is one of the most significant members of the Flaviviridae family, which has four different serotypes (DENV1-4) that only differ by 30–35% of the amino acid sequence (4), and a large number of genotypes within each serotype that varries only by 6–8% nucleotide and 3% amino acid sequence (5). All the variants are infectious and can result in a variety of clinical conditions, from mild dengue fever to severe dengue (6). Every serotype’s primary infection confers lifelong immunity against it, and usually recovers after a self-limiting condition (7). According to the updated dengue case classification, recurrent infection with a heterologous serotype only confers partial or transitory protection, often leading to dengue with or without warning signs (DWS+/-) and severe dengue (SD) (8, 9). Serotype-cross-reactive antibodies may also significantly contribute to the spread of DENV by facilitating viral entry into FcγR-bearing monocytes, a mechanism known as antibody-dependent enhancement (ADE) (10).

The specific antibody response generated after primary infection maintains an immunological memory, and is able to bind and to neutralize a homologous dengue serotype providing lifelong specific-immunity. While in subsequent infection with heterologous serotype, the pre-existing IgG antibodies are able to bind to the heterologous dengue serotype, however, instead of neutralizing the virus, it enhances the infection (11). This impairment in development of robust immune response against heterologous serotype is due to the phenomenon of original antigenic sin (12, 13). The titre of antibodies, targeted towards the virus serotype, are responsible for the primary infection tend to significantly rise and can often remain higher than the levels of antibodies induced towards currently infecting serotype (14).

During recurrent infection, the antibody-mediated amplification of the disease severity sets a high bar to develop a vaccine against DENV. There is currently no such vaccination technique that can completely protect against all four serotypes in naive people (15). The most leading vaccine, a tetravalent live attenuated candidate (CYD-TDV), has received approval in certain dengue-prevalent nations; however, it has the unintended consequence of raising the risk of SD in individuals who have never been infected by DENV (16). As a result, the administration of vaccines, and their development have been limited by the concern that vaccine delivery may put naive people at a high risk of alarming dengue (17).

Nonetheless, several alternative attempts have been made towards the development of safe vaccine with efficient protective immunity and to exempt all risky issues (18). The Laboratory of Infectious Diseases at the National Institutes of Health evaluated a number of monovalent and tetravalent dengue candidate vaccines to determine which had the greatest safety, infectivity, and immunogenicity profiles. TV003/TV005, a vaccine that combines four live attenuated recombinant dengue virus vaccines (rDEN1D30, rDEN2/4D30, rDEN3D30/31, and rDEN4D30), has cleared Phase III clinical trials, and been licenced to a number of manufacturers, including Butantan, VaBiotech, and Merk (19). Furthermore, various additional recombinant tetravalent vaccines (e.g., DEN-80E, TVDV) have passed phase I clinical trials, expressing the prM and E genes of each of the four DENV serotypes from plasmid DNA (18, 20). DENVax candidates, Takeda’s live tetravalent dengue vaccine TDV, and a chimeric dengue-2 PDK-53-based tetravalent vaccination are also undergoing preclinical and clinical trials to stimulate humoral and cellular protective immune responses (21). Most recently a nucleotide-modified mRNA vaccine encoding the membrane and envelope structural proteins from DENV serotype 1 encapsulated in lipid nanoparticles (prM/E mRNA-LNP) was found to induce neutralizing antibody and cellular immune responses in immunocompetent mice. In comparison to a live DENV1 viral infection, this vaccine design displayed serotype-specific protection with little serum cross-reactivity and decreased ADE (22). Since the pre-existing antibodies either raised by the vaccine or the primary infection may increase the disease severity, none of the vaccine candidates are still allowed for human application. Due to the continued absence of a safe vaccine and suitable drugs, other antiviral measures, especially antibody therapeutics, are becoming an attractive option to reduce SD. Many infectious and non-infectious diseases are now being treated with a variety of therapeutic antibodies (23). However, antibody therapeutics against DENV is still challenging due to the ADE effect and higher production cost. Several expression systems and protein engineering options are currently being utilized for the economical production of antibodies and to overcome the biosafety concern of ADE (24, 25).

For the last ten years, our research group has been working on designing and production of monoclonal antibody (mAb) fragments and short peptide vaccines against DENV. The challenges and difficulties observed in developing a safe and effective therapeutic mAb and vaccine motivated us to write this review. Therefore, we explored the NCBI database to find published articles on this topic in the last two decades. In this review, we aim to describe the disease severity, general features, and characteristics of the DENV, along with its surface protein structures, following the different immune targets. Then, we focus on the envelope protein as a potential target in the third part of the review. Here, we deliberate the serotype-specific and cross-reactive epitopes and the therapeutic potential of the neutralizing and cross-reactivity antibodies targeted to these epitopes. After that, the mAbs developed against virus particle-like quaternary structure is described. Lastly, the insights on severe pathogenesis and ADE would provide extended knowledge to design future subunit vaccines and therapeutic mAbs.

## 2 Dengue virus and immune targets

The flavivirus genus includes a group of more than seventy single-stranded, positive-polarity RNA viruses that are mostly spread by arthropod vectors. Most of which are spread by mosquitoes, including the Zika virus (ZIKV), West Nile virus (WNV), Japanese encephalitis virus (JEV), (DENV), and Yellow fever virus (YFV), and some are spread by ticks, which mainly cause Alkhurma diseases, Tick-Borne Encephalitis (TBE), Omsk hemorrhagic fever, and Kyasanur forest disease (26). These viruses can cause a wide variety of illnesses in vertebrates including asymptomatic to moderate fever, flu-like symptoms to fatal encephalitis, arthralgia to severe hemorrhagic fever. Despite genetic and anatomical similarities within the genus or species, infection can have a wide range of clinical outcomes (27). Infection with WNV, JEV, TBEV, Powassan virus (POWV), and ZIKV can result in neuroinvasive infection, whereas DENV, YFV, and ZIKV cause visceral illness (28). Low blood pressure can result from the NS1 protein’s capacity to change vascular permeability. Depending on the flavivirus, lesions may form in the brainstem, cerebral cortex, hippocampus, thalamus, cerebellum, or spinal cord (27). Acute flaccid paralysis (AFP), which is a distinct manifestation of WNV infection, and neuromuscular weakness is seen in 50% cases. Seizures and dystonia are common in JEV patients, and 50% of survivors continue to experience long-term psychological effects (28). Thus, fatality may linger in patients for a very long time and possesses a risk of long-term morbidity. However, there is no antiviral treatment for flavivirus infection, with the exception of certain vaccines against YFV, JEV, and TBEV, and their outbreaks persist until the weather eliminates their vectors (29).

The whole flavivirus genome is approximately an 11-kb RNA with a single ORF that is bordered by the 3’ and 5’ non-coding sequences. This ORF translates 7 non-structural proteins and 3 structural proteins (capsid, membrane and envelope) which are necessary for viral generation. The RNA genome is stored in a structural core of the underlying capsid (C) protein, which is covered on the surface of the mature virion by envelope (E) glycoprotein and membrane (M) protein (30). The envelope protein has to interact with the proper receptor for the flavivirus genome to enter the host cell. The interaction between the envelope (E) protein and the glycosaminoglycans on the surface of the host cell gradually raises the viral concentration over the surface, ensuring tight contact with the receptor (31). Three ecto-domains of the envelope protein, termed as domain I, II, and III, engage with receptors and attachment factors to initiate endocytosis. A conserved fusion loop of domain II, located at the end of envelope proteins, is revealed to be particularly important for starting receptor-mediated endocytosis. In addition, the domain III has also been mapped by the vast majority of strong, neutralising antibodies, underscoring its clinical significance. Evidently, the anti-domain I and anti-domain II antibodies are less effective, nonetheless have greater cross-reactivity, and comprise a large fraction of anti-E IgG antibodies that are now being investigated for therapeutic purposes (32).

The virus that causes dengue fever is carried mostly by female mosquitoes *Aedes aegypti* (Linnaeus, 1762), and to a minor degree by *Aedes albopictus* (Skuse, 1895). The mature form of the virus has a spherical shape with a diameter of 40 to 50 nm, and a single-stranded, positive-polar RNA genome is encased in a bilayer membrane (33). DENV has four distinct serotypes in the Flaviviridae family, DENV1 through DENV4, and a large number of genotypes within each serotype. Infection by any of those can cause a variety of well-defined medical conditions, from mild fever to alarming dengue, which is sometimes associated with a number of fatal complications like plasma leakage, respiratory distress, fluid accumulation, severe bleeding, and organ impairment (34). The serotype and genotype variation may affect the clinical condition differently. For instance, compared to the American DENV-2 genotypes, Asian genotypes tend to produce more serious infections in people (5). According to a research work conducted in Thailand, DENV-2 was most likely to cause DHF (44%) than other serotypes and DENV-4 was less likely (31%) (35). Additionally, the clinical severity varies depending on the interactions between the virus and the host’s immune system. Children who have already acquired immunity to the DENV-1 serotype are susceptible to SD from DENV-2NI-1, whereas DENV-2NI-2B is more virulent in children who have already infected with the DENV-3 serotype (36).

Various phylogenetic studies have reported the presence of recombinant genotype within particular DENV serotype (37, 38). This intra-serotype recombination along with high mutation rate of this RNA virus is responsible for antigenic variation (39). However, the effect of recombination is weaker in creating DENV diversity, and is not driven by natural selection to enhance the fitness of the virus (40). Intra-serotype recombination is a chance phenomenon and cannot solely drive the evolution of a new genotype; however, it may aid the evolution process of DENV towards better viral fitness. Nonetheless, nucleotide variation analysis showed that the occurrence of synonymous mutations was more than non-synonymous mutations in DENV1-4, and mainly found in the non-structural genes which are responsible for viral replication (41). The evolutionary studies provided insight into the processes of positive selection and antigenic diversity in structural and non-structural genes. Therefore, most of the antiviral efforts are concentrating on the conserved areas to avoid diversity.

**Figure 1:**
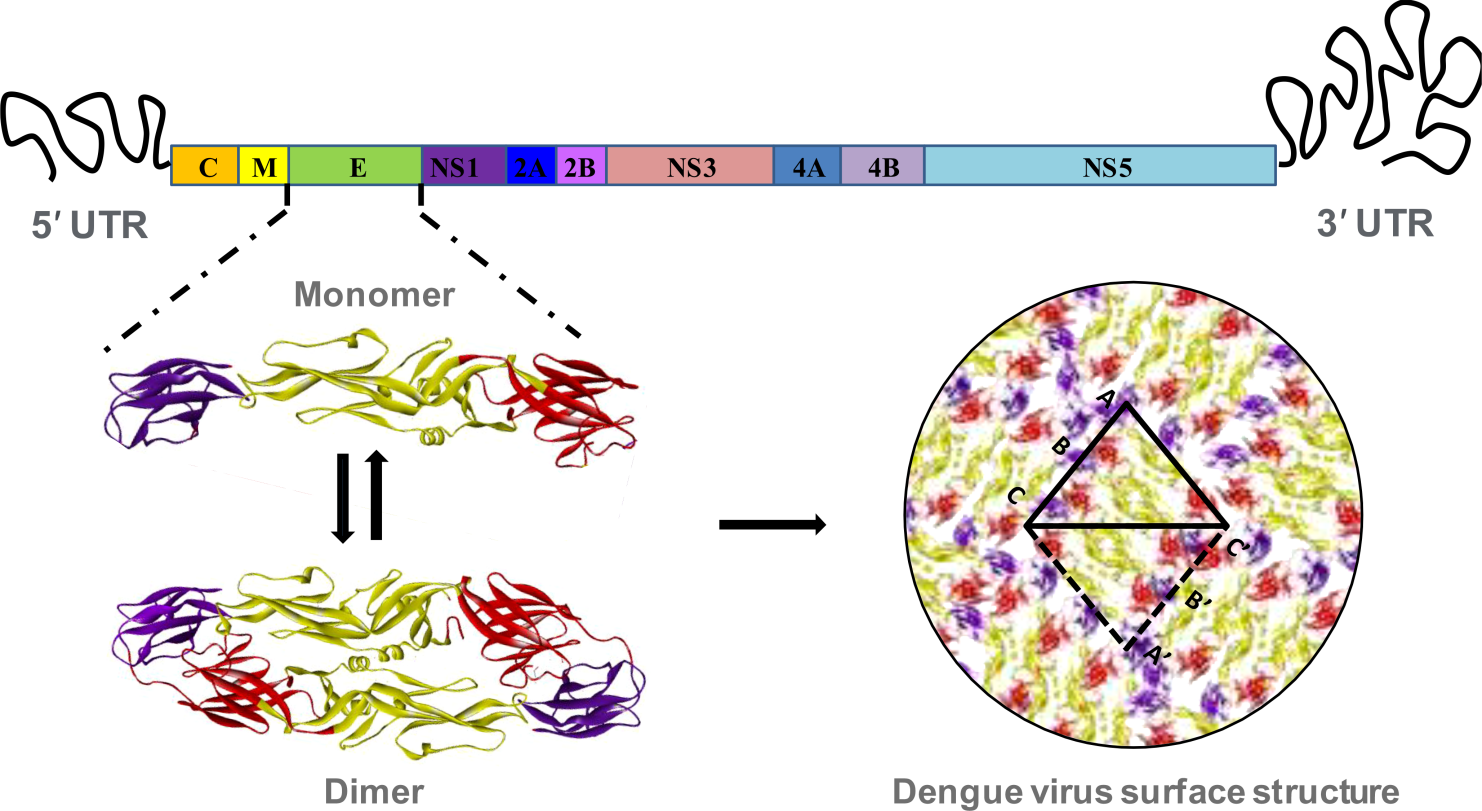
Structural organization of dengue virus envelope protein. The RNA genome of dengue virus is composed of a single ORF, flanking by 5’ and 3’ untranslated region. The ORF includes coding sequence of three structural proteins, and seven non-structural proteins. The envelope (E) is the most significant structural protein of dengue virus surface. The monomer of each of the envelope protein is arranged into head-to-tail homo-dimer and 90 of such dimers are arrange in icosahedral symmetry to constitute the complete surface of dengue virus. Each of the monomeric envelope protein consists of three ecto-domains: EDI, EDII and EDIII. The domain I, II and III are denoting by red, golden and violet color respectively.

**Figure 2:**
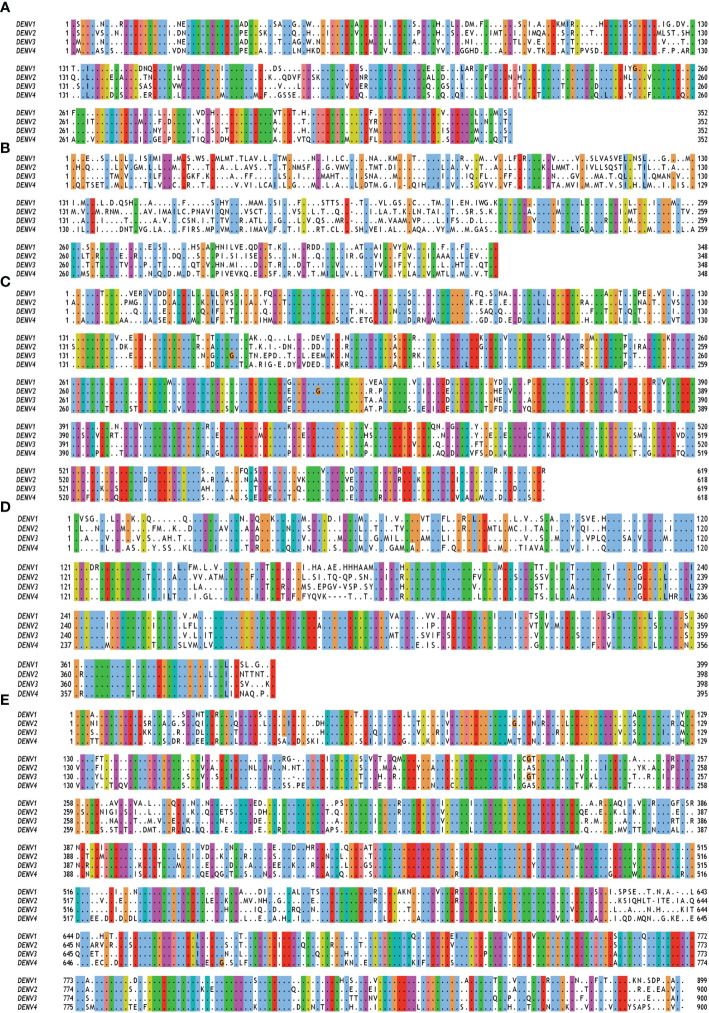
Multiple sequence alignment of non-structural (NS) proteins of DENV serotypes 1 to 4. The amino acid sequences of **(A)** NS1 **(B)** NS2A and NS2B **(C)** NS3 **(D)** NS4A, 2K and NS4B **(E)** NS5 are taken from polyprotein having accession no. QTH36735.1, NP_056776.2, YP_001621843.1, NP_073286.1 for DENV serotypes 1 to 4 respectively. Fully conserved amino acids are indicated with black dots while variable amino acids are displayed in the Clustal Omega format using Jal view. As per the Global Similarity (BLOSUM62) analysis, the sequence similarity index for NS1, NS2, NS3, NS4 and NS5 proteins are 0.77 ± 0.03, 0.51 ± 0.04, 0.82 ± 0.03, 0.75 ± 0.02, and 0.79 ± 0.02, respectively.

The DENV is generally spherical in shape, and is made up mostly of a 10.7 kb RNA, capsid proteins, and envelope proteins. It enters into the host cells by interacting with cell surface receptors followed by clathrin-mediated endocytosis and trafficking into the late endosomes. The low pH condition of the late endosome triggers structural rearrangements of the DENV E protein and promotes fusion of viral and host membranes (33). Once viral genome is released into host cell cytoplasm, viral RNA is translated into a single polyprotein that is subsequently cleaved into three structural and seven non-structural proteins (NS1, NS2A, NS2B, NS3, NS4A, NS4B, and NS5) by viral (NS3) and host proteases (**Figure 1**). The capsid (C), membrane (M), and envelope (E) proteins give the structural shape, and the non-structural proteins are engaged in virus multiplication (23). These non-structural proteins show different levels of sequence variability among different serotypes. NS2 proteins show the highest level of variability among all the non-structural proteins (**Figure 2**). These non-structural proteins direct RNA replication and packaging. Negative-strand viral RNA is synthesized first that directs positive-strand RNA synthesis. Progeny RNA further associates with C proteins to form a nucleocapsid which buds into the ER to acquire lipid membrane containing heterodimers of E and prM proteins. The immature assembled virus is transported through Golgi apparatus where prM protein is cleaved by furin. After release into extracellular space, the cleaved pr peptide is dissociated and virions become fully infectious (42, 43).

The complete RNA genome is enclosed in a nucleocapsid core, which is surrounded by a lipid bilayer membrane. The outer surface of the membrane also remains covered with the E protein. There are 180 copies of E proteins which are arranged into 90 head-to-tail homo-dimers, and three of such homo-dimers are arranged in a raft with two asymmetric units. These 30 units are finally organized in an icosahedral symmetry (**Figure 1**) (44). There are three domains in the envelope protein, such as domain I, II, and III. Although domain I belongs at the N-terminal end, in the three-dimensional structure it is located in the middle, hanging domains I and II on their opposite sides. In addition, domain II has two extended loops, including a conserved fusion loop at one end of the E protein structure and a neighbouring bc loop. Whereas domain III, an immunoglobulin-like structure at the other end of the molecule, is thought to connect with the host cell surface receptor to induce endocytosis (45). As the virus enter in a host cell endosome, the acidic condition causes envelope proteins to undergo conformational changes, causing fusion loops to produce trimeric spikes that penetrate the endosomal membrane. This rearrangement of the trimeric envelope protein enables the release of the viral RNA into the host cell cytoplasm by merging the viral membrane with the endosomal membrane (46).

## 3 Antibodies targeting the DENV envelope protein

The mAbs have certain unique advantages in dengue treatment as compared to the polyclonal antibodies. Neutralization escape mutations might be prevented by targeting numerous epitopes at a time to neutralize DENV (9). Nonetheless, weak or sub-neutralizing antibodies may induce ADE, a phenomenon most often associated with DENV infection (47). The major fraction of antibodies against DENV are not neutralizing, and the use of polyclonal antibody preparation carries a significant risk of ADE. Therefore, highly specific neutralizing mAbs are thought to be produced *in vitro* in a larger quantity, and the passive immunization with these antibodies can significantly reduce the DENV associated severity (9).

**Figure 3:**
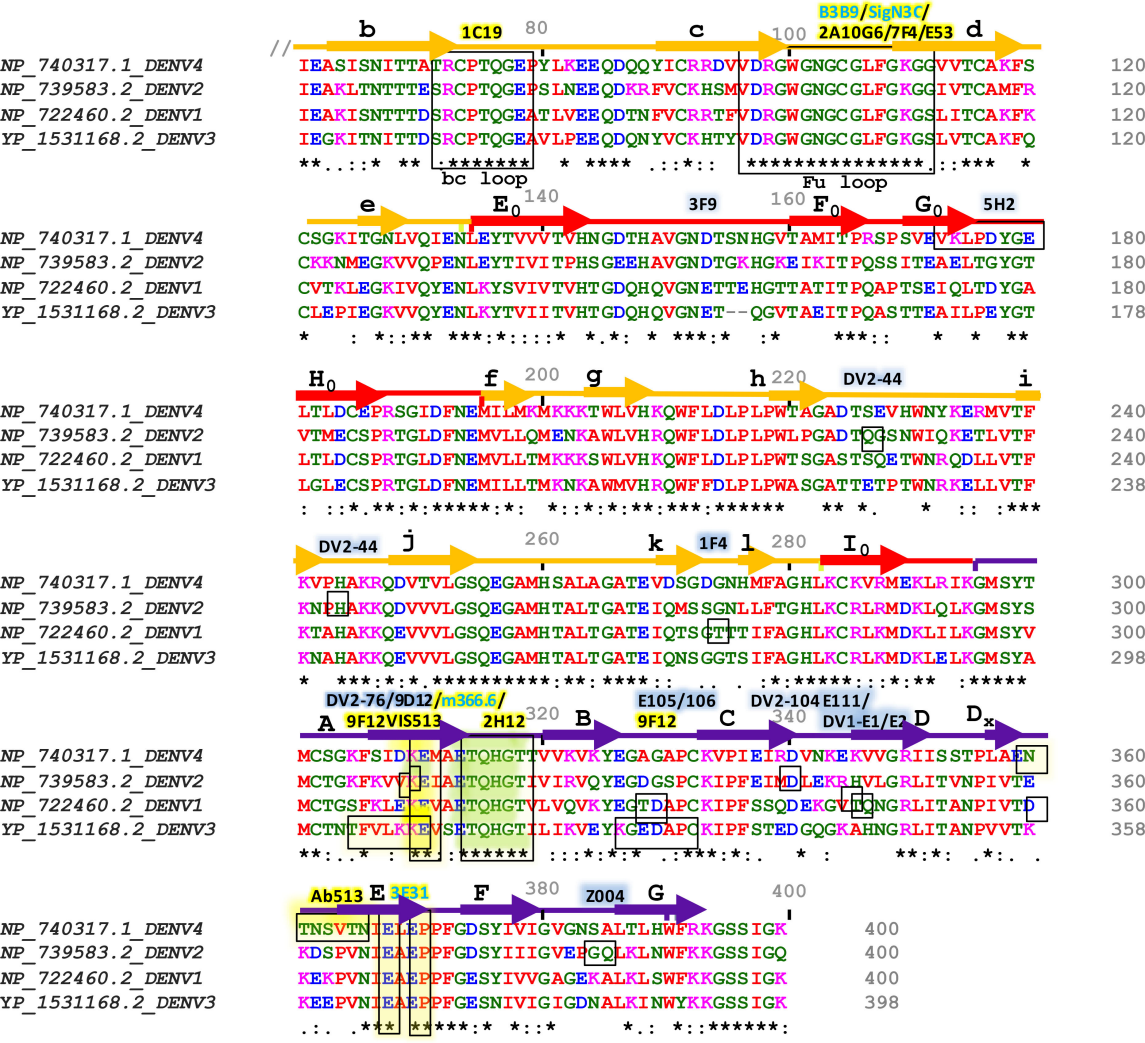
Sequence alignment of Envelope proteins ecto-domains (60 to 400 residues) of four dengue serotypes. Fully conserved amino acids are indicated with black stars (*) at the bottom of each row. Domains I, II, and III are schematically indicated with red, yellow, and blue color bars. Secondary structures are shown above the amino acid cluster (arrows indicate *β* strands, and the lines indicate helices). Each of the *β* strands of domains I, II, and III have been denoted with (A_0_ to H_0_), (a to l), and (A to G), respectively. The interacting residues of serotype-specific and cross-neutralizing antibodies are marked with blue and yellow shaded boxes, respectively, and their corresponding antibodies are mentioned above the bars with the same color-shed. The antibodies which were identified without any ADE effects are shown with blue letter code name and mentioned above the sequence alignment.

The outer surface of DENV is made up of 180 copies of a monomeric envelope protein that is arranged with icosahedral symmetry (44, 45, 48) and is widely recognized as a target of neutralizing antibodies (49). Since the amino acid sequence of E proteins being 72% to 80% similar across all four serotypes, DENV neutralizing antibodies may either be type-specific or cross-reactive (**Figure 3**) (50). Structural studies reveal that each E subunit consists of three distinct β-barrel domains termed as domain I, II, and III. Two well-characterized hydrophobic loops (fusion and bc) are present at domain II and a receptor-binding site at domain III (51). The primary repertoire of antibodies in human serum targets the fusion and bc loop of domain II, which are identified mostly as cross-reactive but poorly neutralizing (52, 53). A minor repertoire of antibodies is found to target domain III, and they are highly neutralizing to some specific serotypes but not equally effective against all the four serotypes (51). Therefore, we have categorized the antibodies mainly into two groups: serotype-specific and cross-reactive; and also, have shown their target site specific critical response along with the ADE effects (**Table 1**). Besides, a small number of mAbs have been identified for interacting with different structural residues of domains I, II, and III (79, 85), some of which are found serotype-specific, and some are cross-reactive; therefore, they are further categorized as quaternary epitope-specific antibodies.

**Table 1:** DENV specific antibodies with their corresponding target epitopes, key interacting residues, neutralizing effects and ADE effects.

Antibody	Target Virus	Epitope	Key interacting residues	Neutralizing effects	ADE effects	References
Antibodies targeting serotype specific epitope						
**DV1-E1 & E2**	DENV1	DIII lateral ridge	LR(Q347, N348 & D360)	Neutralizes only DENV1	NA	(54)
**E106**	DENV1	DIII lateral ridge	A(K307,K310);B(K325,Y326); BC(E327,T329,D330);K361,362	Neutralizes 5 DENV-1 genotypes	NA	(55)
**E111**	DENV1	DIII & CC’ loop	N-terminal (300,301); C strand, CC’ loop (E345), C’ strand (334–351); EF loop (372);FG(382-384)	Neutralizes DENV-1 genotype 2	NA	(56)
**E105/E106**	DENV1	DIII Lateral ridge	BC(G328, T329 & D330), DE (K361 & E362), & FG (K385)	Neutralizes 5 DENV-1 genotypes	Not tested	(57)
**1A1D-2**	DENV2	DIII Lateral ridge	LR(K310) E383, P384	Strongly neutralizes DENV2	NA	(58)
**9D12**	DENV2	Lateral ridge	LR(K310) E383, P384	Moderately neutralizes DENV2	Not tested	(58)
**3F9**	DENV2	DI centre	DI-centre	Neutralizes DENV2 serotypes	NA	(59)
**DV2-44, 76, 104**	DENV2	DI & DII, DIII	DI & II(K88, Q233, H244); DV2-76_DIII(V309); DV2-104 _DIII(P334, M340,H346)	Neutralizes diverse DENV2 strain	Prevent ADE	(51)
**5H2**	DENV4	DI (F0, G0 and H0 and G0H0)	DI(E172, K174, P176, D177, E180, R293)	Neutralizes DENV4	NA	(60)
**1G6**	DENV4	EDIII	EDIII (387-ALTLH-390) T388, H390	Neutralize DENV4	Not mentioned	(61)
**3H2**	DENV-2	EDIII	DIII-DI hinge residues (298–303) BC loop (328–332), K361,V282, E383, P384 & C strand, CD loop	Neutralizes DENV2	Marginal ADE	(62)
**2C8**	DENV-2	EDIII	DIII-DI hinge residues (298-303) BC loop (328-332), K361,V282, E383, P384	Neutralizes DENV2	Induce ADE	(63)
**DENV-290**	DENV-2	EDII	EDII	Neutralizes DENV3	Not mentioned	(62)
Antibodies targeting cross-reactive epitope						
**7F4**	DENV-1,2,4	EDII	DII (E67, E69 and E118)	Cross-reactive but neutralizes DENV1	Undetected	(46)
**E53**	WNV, DENV1-4	EDII fusion loop	WNV_FL(G104, C105, G106, L107, G109,K110); bc(C74, T76, M77, G78, E79)	Cross-reactive	Prevailing ADE	(64)
**m366.6**	DENV1-4	DIII A strand	DIII_A(K310)	Cross-neutralizing	Undetectable level	(65)
**3E31**	DENV1-4	DIII	AB (314–370), E(365-370), Q316, H317, E368, E370	Cross-neutralizing	Does not promote	(66)
**Ab513 (4E5A)**	DENV1-4	DIII	DE(358-365),Y360, S363	Broadly neutralizing	Undetectable level	(67)
**VIS513 (4E11)**	DENV1-4	DIII A strand	DIII_A(K310, E311)	Cross-neutralizing	Minor level	(68)
**2H12**	DENV1-4	DIII A strand	A(314-317); FL(K110, E114, T115, Q116, H117)	Cross-neutralizing except DENV2	Minor or negative	(63)
**9F12**	WNV, DENV1-4	DIII A strand	A(K305,K307, K310); B(327-331)	Cross-neutralizing	Not mentioned	(69)
**N297Q-B3B9**	DENV1-4	DII fusion loop	Fusion loop	Cross-neutralizing	Lack of ADE	(70)
**SigN-3C**	DENV1-4	DII fusion loop & DIII	FL(G100, W101); EDIII(K310)	Cross-neutralizing	Abrogates ADE	(71)
**2A10G6**	DENV1-4	DII fusion loop	ZIKV, DENV1-4 FL(98D,99R,100X,101W)	Cross-neutralizing	Undetected	(72, 73)
**1C19**	DENV1-4	DII-bc loop	DII-bc(R73,G78,E79)	Cross-neutralizing	Undetected	(74)
**d448**	DENV1-4	DII and M	DII (D215, P219, M237, Q256, and G266)	Cross-neutralizing	Undetected	(75)
**DM25-3**	DENV1-4	VLPs	DII-FL(W101)	Cross-neutralizing	Undetected	(76)
**Z004**	ZIKV DENV1	DIII lateral ridge	LR(P384, G385)	Neutralizes ZIKV and DENV1	Not tested	(77)
**MZ4**	ZIKV DENV2	DIII & DI linker	ZIKV(residues 299-306)	Neutralizes ZIKV and DENV2	Lesser extend	(78)
Antibodies targeting quaternary epitope						
**HM14c10**	DENV1	Hinge region between DI- DII & DIII	DI(I161, T165, Q167,E172); DII(S273, G274,T275,T276); DIII(E311,E309,E327)	Potentially neutralizes DENV1	Not mentioned	(79)
**5J7**	DENV3	DI–DII HR & DIII, FL loop	DI & DII (E123, E126, HR-Q269, N270) EDIII(K307, K308)	Neutralizes DENV3	Not mentioned	(80)
**1F4**	DENV1	DI & DI-DII HR of one ED1 and DIII of ED2	Doa; Eo, EoFo loop, Fo (155–165), Go strand, GoHo loop (170–177), and kl loop (272–276)	Strongly neutralizes DENV1	Not mentioned	(81)
**2D22**	DENV2	Dimer of DIII; and DII	DIII(R323) & EDII (FL and bc loop)	Neutralizes DENV2	Prevent ADE	(82)
**1L12**	DENV2	DIII	DIII(R323)	Neutralizes DENV2	Not mentioned	(59)
**A11, B7, C8, C10**	DENV2	Dimer of EDI & EDII	DI(148-159, N153 glycan); DII(67-74, N67 glycan), FL loop (97-106), ij loop (246-249)	Neutralizes DENV1-4	Not mentioned	(83)
**J8, J9**	DENV1-4	DI	DI (K47,H149, V151, F279)	Cross-neutralizing	No ADE	(84)

NA, Not Available.

### 3.1 Antibodies targeting serotype-specific epitopes

**Figure 4:**
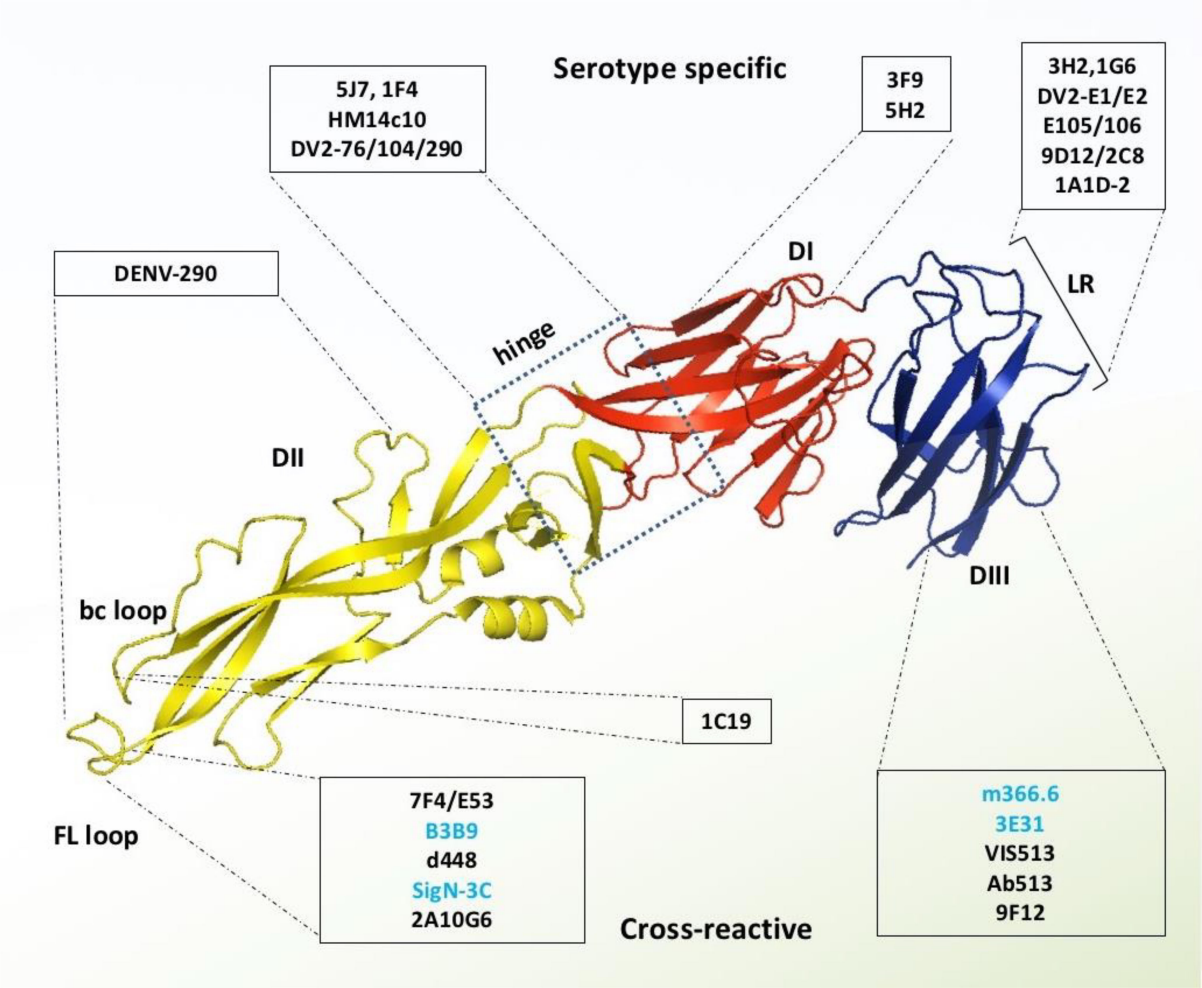
Monoclonal antibodies targeting DENV E protein. Domains I, II, and III are schematically indicated with red, yellow, and blue colors, respectively. Antibodies in the light blue shed are serotype-specific that target mainly domain I, hinge between domain I and II, and lateral ridge of domain III. In contrast, the antibodies in the light-yellow color are cross-reactive and target mainly FL and bc loop of domain II and A strand of domain III. The antibodies which were identified without any ADE effects are shown with blue letter code name.

Several research groups are currently focusing on the therapeutic potential of type-specific antibodies, because they are examined as highly neutralizing, and provide persistent immunity against homologous DENV serotypes (9, 58). Since DENV1 is the most dominant type of the four serotypes (DENV1-4) to cause infections, DENV1-specific antibodies have been prioritized for therapeutic studies. Two of such DENV1 specific mAbs (E1 and E2) have been reported which exhibit strong neutralizing potency, but have no cross-reactivity to other serotypes. Further, structure-guided epitope mapping reveals that these strong inhibitory antibodies bind with a unique epitope between Thr346 and Asp360 on the lateral ridge of DENV1’s domain III (**Figure 4**). Mutational study and sequence analysis identify that Gln347 and Glu360 residues are critical for inhibition of DENV1 infection, and these residues are highly conserved among most of the DENV1 genotypes, whereas diverse from DENV2-4 (54).

Similarly, E105 and E106 antibodies bind potentially with all five DENV1 genotypes and exhibit strong neutralizing activity (57), however, their binding sites are distinct from E1 and E2 binding sites in domain III. Epitope mapping reveals that both of these antibodies lose their binding activity after the alteration of residues Gly328, Thr329, and Asp330 in the BC region, Lys385 in the FG loop, and the substitution of Lys361Glu and Glu362Lys in the DE region of domain III’s lateral ridge area (57). Protein’s crystal study divulges that the contact residues of the E106 binding site are mostly conserved among the five genotypes of DENV1 but vary with other serotypes. Therefore, mAbs, E105 and E106, neutralize all the DENV1 genotypes, nonetheless, it shows poorly detectable neutralizing potential against other serotypes (55, 57). Several studies have shown that the domain III lateral ridge specific mAbs are not influenced by temperature or time-dependent structural changes, whereas the neutralization efficiency of domain III specific mAbs, E105 and E106, are found to be enhanced with the increment of temperature and incubation period (56).

Another mAb has been reported which distinguishes between distinct DENV1 strains by neutralizing a cryptic epitope in domain III. The domain III of DENV1 has a non-conserved CC’ loop that mAb E111 binds to and is anticipated to be inaccessible on both immature and mature virions. Nonetheless, it is capable of neutralizing some of the DENV1 genotypes (56). Therefore, it is assumed that the epitope recognition and virus neutralization by the E111 antibody may happen independently to the temperature, incubation time, and the epitope residues. In other words, the structural alteration of the virus surface influences the neutralization by binding antibodies with a more or less exposed epitope in a strain-dependent manner (86). Therefore, it is suggested that the sequence variation within CC’ loop and their surrounding binding sites are responsible for such differential behavior of these antibodies with various DENV1 genotypes (56).

The second most serotype-specific neutralizing mAbs have been identified against DENV2, which binds with complex structural epitopes of domain III that overlap between A strand and neighbouring lateral ridge residues. Two of such sub-complexes targeting antibodies are 9D12 and 1A1D-2; 1A1D-2 is a firmly, and 9D12 is a moderately neutralizing antibody (58). Their tertiary binding sites are highly conserved, and share residues of both the cross-neutralizing A strand epitope, and serotype-specific lateral ridge epitope. Further epitope mapping reveals that this kind of specificity is determined by the centre residues Glu383 and Pro384 in the FG loop, that are conserved among various DENV2 strains, but diverse in other serotypes (58). Whereas, the residues Lys305, Lys307, and Lys310 of A strand interact with the sub-complex specific mAbs, which share binding residues of cross-neutralizing mAb 4E11 (87). Among these three residues, the Lys310 is totally conserved in all the serotypes, and Lys305 and Lys307 are partially conserved, only between DENV4 and DENV1 strains (**Figure 3**). Therefore, it suggests that their distinct binding specificities and neutralizing efficiency to DENV2 strains are due to the precise interactions with the specific lysine residues on the A strand and two centre residues (Glu383 and Pro384) in the FG loop.

Furthermore, mAb 5H2 was identified from chimpanzee, can potentially bind to the DI of the DENV4 envelope protein, and can potentially neutralize it. Crystal-structure studies reveal that the residues of 5H2 Fab fragment form a hydrogen bond with the side chains of epitope residues, Glu172 and Lys174 of G_0_ strand, Asp177 of G_0_H_0_ loop, Glu180 of H_0_ strand, and Arg293. Except for the Glu172, all the epitope residues that engage in hydrogen bonding are variable across all the DENV serotypes (**Figure 3**). However, the 5H2’s key binding residues are conserved among all of the sequences of the DENV4 strains available in the database (60). Further investigation reveals that the 5H2 Fab antibody binds with the linker residue Arg293 (between DI and DIII), thereby preventing the conformational change of fusogenic event. Locking these two subunits in the pre-hairpin trimer ultimately blocks the membrane fusion into the endosome (60, 88). Another DENV4 envelope domain III specific neutralizing mAb, 1G6, plays role at one step before the fusogenic event. Pre- and post- adsorption inhibition experiment reveals that the 1G6 antibody prevents viral adsorption at the primary stage of life cycle (61). Since domain III engages in binding with many cell receptors (89), the binding of 1G6 antibody with domain III may block the host cell receptor binding as well as potential endocytosis.

Serotype-specific mAbs have also been identified against other regions of DENV envelope protein such as the epitopes located especially at the centre of domain I, hinge region between domain I and II, dimer interface of domain I and II, fusion and bc loop of domain II, lateral ridge, CC’ loop, and the A strand of domain III (57, 58) (**Figure 4**). For instance, mAb DV2-48 and DV2-51 bind primarily with the key residues Gly177 and Glu184, respectively of the domain I epitope, in the DENV2 envelope. These binding sites are similar to the binding site of DENV4 specific protective antibody 5H2, which targets key amino acid residue Lys174 in DI (61). There are three more antibodies, DV2-44, DV2-76, and DV2-104, which are also capable of neutralizing DENV2 strains without inducing ADE (51). Epitope mapping shows that DV2-44 interact with the residues Lys88, Asn233, or His244 in the dimer interface of domain I and II, whereas DV-76 loses binding due to the mutation of conserved residues Lys305, Lys307, and Val309 in the A strand of domain III; which are similarly targeted by the sub-complex specific antibodies 9D12 and1A1D-2 (51, 58). Unlike the domain III lateral ridge-specific mAb targeting FG loop (especially the Glu383 and Pro384 residues), DV2-104 interacts with a conserved residue Pro336 located on the bridge between BC loop and C strand, and also two additional DENV2 specific residues Met340 and His346, resided respectively in the C strand and CC’ loop (44, 51). Due to the differential binding specificity, these antibodies so far provide protection exclusively against the DENV2 serotype.

Generally, most serotype-specific DENV antibodies induce ADE at a sub-neutralizing concentration (90). Nevertheless, recently two unusual DENV2 type-specific mouse mAbs, 2C8 and 3H5, were characterized to domain III of the DENV2 envelope protein, which are potentially neutralizing; however, they significantly differ in their ADE properties. Analysis of their crystal structures revealed that 3H5 binds with some extra amino acids in the C strand and CD loop compare to 2C8. The crucial interacting residues Lys344 and Arg345 are situated in close proximity to the viral membrane and remain hidden in the inter-dimeric envelope structure. Due to this differential binding, 3H5 exerts strong neutralizing potency with low or negligible ADE, whereas 2C8 possesses a similar neutralizing capacity but promotes a higher level of ADE (62). The target epitope and the functional properties of 3H5 are symmetrical to the previously described antibody DV2-104 that also strengthen the explanation about their specificity and the reasons for their strong neutralizing potency without ADE.

Lastly, 15 more human mAbs have been reported, some of which shows efficient neutralizing activity against DENV3, although their neutralization potency varies from 10 to 15-fold across different genotypes. These antibodies were categorized into three major subsets viz. group 1 mAbs, targeting domain I epitopes (DENV-437, -286, -298, -354, -404, -406, -443); group 2 mAbs, targeting domain II epitopes (DENV-115, -290 and-419); and group 3 mAbs, targeting domain III epitopes (DENV-66, -144). Group 1 mAbs poses both modest and robust neutralizing activity *in vivo*, as was the case with DENV-443 and -298 respectively. Whereas DII specific group 2 mAbs mostly reduce *in vivo* viral load, and among these, DENV-290 is the best neutralizing candidate (62). Therefore, it suggests that in addition to viral genotype, selection of an appropriate target site is also crucial for protective immunity.

### 3.2 Antibodies targeting cross-reactive epitopes

Most of the DENV envelope protein-specific mAbs are serotype-specific, and targets mainly the domain III epitopes (57, 58). However, a number of cross-reactive mAbs have been identified, which exclusively target the fusion and bc loop of domain II; and a small fraction of them are also found to target some of the conserved epitopes in domain III (**Figure 4**).

In general, domain II specific anti-E antibodies constitute the majority of cross-reactive anti-E antibodies identified from dengue patients. These antibodies have low neutralizing efficacy but are extremely cross-reactive with many serotypes, even comparatively more cross-reactive than domain III specific mAbs (91, 92). A significant fraction of them is concentrated on the evolutionary conserved fusion (FL) and bc loop of domain II, and capable of neutralizing all the four DENV serotypes. Sometimes the fusion loop specific antibodies are bifunctional, having both neutralizing and enhancing activities. For instance, a fusion loop specific human mAb, D23-1B3B9 (B3B9) is strongly neutralizing against all of the DENV serotypes (70, 93). However, it shows viral enhancing activity in FcγR bearing cells at a sub-neutralizing concentration that further limits its therapeutic trial. Despite the fact, this antibody’s chimeric form (N297Q-B3B9 rIgG) has *in vivo* neutralizing effectiveness against all four serotypes without viral boosting activity (70, 94). Another fusion loop specific mAb, 2A10G6, is cross-reactive with DENV1 to 4, ZIKV, JEV, TIEV, YFV, and WNV; and potentially neutralizes all DENV strains, ZIKV, YFV and WNV. Structural and functional studies reveal that 2A10G6 bind with a conserved motif 98DRXW101 of the domain II fusion loop and confers protection against the lethal doses with DENV1-4, WNV, and ZIKV viruses in the mouse model (72, 73). Further functional tests show that this antibody prevents infection at a stage of the DENV life cycle after attachment.

Recently, a potent cross-neutralizing antibody SIgN-3C has been identified, which targets a novel dimeric epitope consisting fusion loop of domain II and III. Alanine scanning and structural study reveal that SIgN-3C binds with a conserved dimeric cluster of DENV envelope protein comprising residues domain II (G100, W101) in one monomer and domain III (K310, R323) in another monomer. Importantly, the SIgN-3C and its LALA-variant (substitution mutations from leucine to alanine at positions 234 and 235) are cross-reactive and equally effective with *in vitro* and *in vivo* protection against all of the DENV serogroups, therefore it seems to escape the ADE danger (71). Although VIS513 and 1A1D-2 bind with a similar epitope in domain III, they are not equally potent in specificity and *in vivo* neutralization (68, 71). Consequently, it implies that invariant residues of the fusion loop and domain III are crucial for maintaining the structural integrity of the virus and responsible for inducing cross-neutralizing antibodies.

On the other hand, an anti-fusion loop antibody (E53), which predominantly concentrates on the immature form of the dengue and West Nile viruses. Compare to other anti-fusion loop antibodies, E53 neutralizes partly mature viruses but not completely mature viruses (95). X-ray crystallographic studies reveal that E53 interacts with twelve residues, comprising the fusion loop (Gly104, Cys105, Gly106, Leu107, Gly109, and Lys110) and the neighboring bc loop (Cys74, Pro75, Thr76, Met77, Gly78, and Glu79) residues of domain II. Further functional studies demonstrate that E53 inhibits furin-catalyzed prM processing in mild acidic pH and prevents the maturation of the immature virus particle (64). Despite preserving cross-reactivity among all DENV serotypes and WNV, E53 can still induce ADE and increase disease severity by rendering the immature or partially mature form of DENV particle. Therefore, to exempt ADE, an *in silico* designed scFv mutant variants were developed using Z-docking and molecular dynamic simulation (96). *In vitro* binding study reveals that some of these scFv variants possesses nano-molar (nM) range affinity towards the recombinant Fu-bc subunit protein (97, 98). Another fusion loop specific mAb ‘DM25-3’ reacts more potentially with mature virus-like particles (mD2VLP) than immature virus-like particles (imD2VLP). Further, epitope mapping reveals that conserved Trp101 residue in the fusion loop is critical for its proper binding and neutralization. Hence, it anticipates that some residues, like Trp101 of the fusion loop, remain exposed at the mature stage, which facilitates its binding with mature form of virus particle. In contrast, E53 can only attach while virion particles go through a structural shift brought on by low pH at the “breathing” stage and the fusion loop gets fully exposed (76, 95).

Moreover, a human mAb, 1C19, recognizes the invariant region of bc loop, located at close-proximity of the domain II fusion loop, and exhibits ultra-high neutralizing potency for all the four DENV serotypes (74). Since some of the anti-fusion loop antibodies, such as E53 and 2J21, also bind with the adjacent bc loop residues, probably due to this reason, these antibodies may block the 1C19 binding site and prevent more potent bc loop specific immune response (58, 74). Therefore, a high dose of 1C19 application might compete for binding with fusion loop specific antibodies and thereby suppressing fusion loop-mediated virus transmission, resulting in a higher level of neutralization potency. Alternatively, an scFv variant of IC19 antibody has been developed and synthetically engineered in *the Aedes aegypti* genome. The resulting homozygous mosquitoes are found to complete refractoriness to DENV infection, and thus it appears as a novel anti-dengue strategy (99).

Despite targeting fusion and bc loop of DII, some of the cross-reactive antibodies target other conserved residues in DII. Among these, antibody d448 interacts with five crucial residues (Asp215, Pro219, Met237, Gln256, and Gly266) that are located in the hidden junction between the membrane and the ecto-domain of envelope proteins. As these residues occupy a crucial structural location, it is possible that binding of the D448 antibody to the important structural component of the DENV coat protein may interfere with the membrane-envelope interaction and prevent “breathing” during viral maturation (75).

The exact role of antibodies in the prevention of recurrent DENV infections and increase the disease severity is still unknown. Most of the neutralizing mAbs against DENV1 to 4 show enhancing activities at sub-neutralizing doses (90). This bilateral phenomenon further discloses that each of the DENV antibodies has two distinct roles; one is neutralizing, and another is enhancing depending on its available concentration. Paradoxically, 3H12 only displays promoting activities even at higher concentrations whereas 7F4 displays neutralizing abilities at high IgG levels but no increasing activities even at lower values. Additionally, a IgG3 subclass of 7F4 antibody recognizes a novel epitope near the glycan moiety of 67 asparagine on the domain II of the envelope protein (100), which has a neutralizing potency of 10- to 1,000-fold stronger than any other previously reported humanized mAbs targeting either DI or DII. However, the IgG1 subclass of 3H12 exhibits only enhancing activities, which targets epitope other than DI and DII (76). Besides, mAb 15C12 primarily recognizes the A strand residue Glu309 in domain III, has both neutralizing and boosting capabilities (46). Therefore, it suggests that the neutralization potency of an anti-DENV mAb not only depends on its available serum concentration but also depends on the target epitopes and the antibody sub-types.

A small number of domain III specific antibodies are also found to cross-reactive and capable of neutralizing all the four DENV serotypes with minor or no antibody-mediated virus enhancement. One of these cross-neutralizing antibodies, 3E31, identifies a thermo-sensitive, invariant epitope in domain III (101). X-ray crystallographic analysis reveals that it binds residues in the AB loop from positions 314 to 319, the β strand from positions 365 to 370, as well as residues at positions 321, 323, and 352. Moreover, its Fab format forms H-H bonds with the residues Gln368, His317, and Glu370, which are remarkably conserved from serotype DENV1 to 4 (**Figure 4**). Reportedly, mAb 3E31 neutralizes all the dengue serotypes by inhibiting envelope-mediated membrane fusion, which sterically hinders the trimer formation during receptor-mediated endocytosis. Unlike the anti-fusion loop antibodies, 3E31 does not induce viral amplification even at the minimum-neutralizing level (66).

Similarly, a mouse mAb, 2H12, can cross-react with all of the DENV serotypes and also neutralizes all except DENV2 with or without a minimal ADE. Co-crystallization study of 2H12 Fab antibody with recombinant domain III reveals that it shares the highly conserved epitope (residues 314-317) in AB loop with 3E31 binding site that usually resides buried in the mature form of virion; however, at the post-antibody binding state, it undergoes rearrangement of overall surface conformation. Furthermore, this binding is characterized as temperature-sensitive, and the neutralizing potency is significantly inadequate toward DENV2 at lower temperatures compared to the other serotypes (63). Moreover, another mouse antibody, 9F12, cross-reacts with all DENV serotypes even with WNV; and neutralizes all four serotypes in plaque reduction assays. Further structural and functional studies reveal that this antibody binds with nano-molar affinity to the conserved epitope of domain III that comprises residues of Lys305, Lys307, Lys310, and Gly330 present in the AB loop. Adsorption and fusion assays divulge that 9F12 neutralizes all DENV serotypes by inhibiting the early fusion steps; however, the post-exposure ADE responses are still unknown (69). Despite sharing a common antigenic part in the AB loop with 2H12 and 9F12, 3E31 is still unique as it does not promote ADE, and so far, it is due to its additional binding residues.

On the other hand, two murine antibodies, 1A1D-2 and 4E11, bind with an overlapping epitope including A and G strands residues of domain III (102). Both of the antibodies bind and strongly neutralize all three DENV serotypes except serotype 4, although, 4E11 provides intermediate level protection against it. Structural study reveals that the difference in the neutralization scheme of these two mAbs is due to a shift in the epitope of domain III; the epitope shift is towards the A strand in the case of 1A1D-2, whereas the epitope is located at the center of A/G strands in the case of 4E11. Therefore, 4E11 antibody has been modified to increase its binding potency toward the domain III of DENV4 (103). One such engineered scFv variant is Ab513, which has increased affinity and neutralization potency at least 75-fold for the DENV4 compare to 4E5A, an early version of the engineered 4E11 antibody (104). In addition to DENV4, the resulting variant can also neutralize all three serotypes (DENV1-3) with EC50 values less than 200 ng/ml (67). Another 4E11-based engineered antibody, VIS513, targets the exposed region of domain III, overcoming antibody-enhanced infection (70). Structural study indicates that mutation at the conserved Glu311 with Asp311 may decrease VIS513 binding by disruption of hydrogen bond and van der Waals interactions. Preclinical research reveals that pre- and post-peak viremia treatment with VIS513 led to neutralize all the DENV infections, although RNAemia remains detectable at post-treatment in non-human primates (NHPs) (105).

Recently, another mAb, m366.6, targeting domain III, has been isolated from a human naïve antibody library and is capable of neutralizing all four DENV serotypes *in vitro*, and protecting a DENV-infected mouse model without causing any detectable ADE effect. Computational docking study reveals that unlike serotype-specific antibody 1A1D-2, m366.6 interacts with key epitope residue K310 with CDR-L1 rather than CDR-H1, which explains why m366.6 is cross-reactive despite sharing a similar epitope with serotype-specific mAb 1A1D-2 (**Figure 4**) (65).

In addition to inter-serotype cross-neutralizing mAbs, a novel group of DENV/ZIKV cross-neutralizing antibodies has been identified. One such example is Z004, which targets the lateral ridge of domain III and potentially neutralizes both ZIKV and DENV but no other DENV serotypes (77). Similarly, MZ4 is also a potent ZIKV/DENV cross-neutralizing antibody which targets a novel epitope positioned at the linker between DIII and DI. It was found exceptionally protective against both of the deadly ZIKV and DENV2, for which IC_50_ values are lesser than previously identified any other ZIKV and DENV2 specific mAbs (78). Since MZ4 shares the binding site (domain III and I linker) with previously described chimpanzee 5H2, it suggests that MZ4 may prevent the structural rearrangement during the fusogenic event and blocks membrane fusion as like 5H2 (88, 102).

### 3.3 Antibodies targeting quaternary epitopes

**Figure 5:**
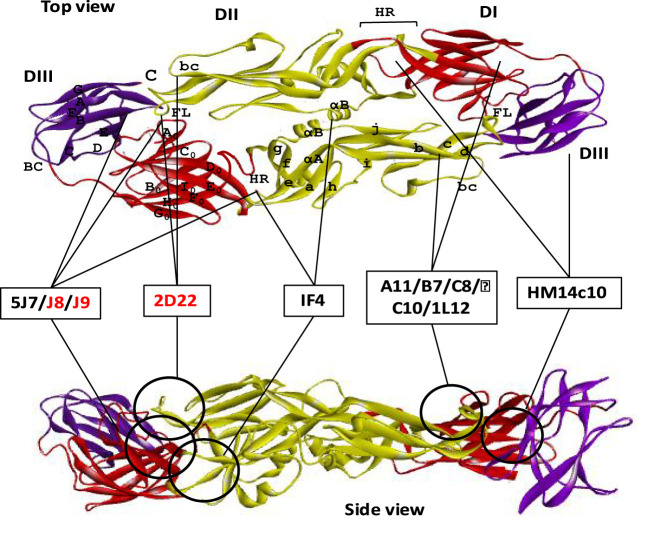
Neutralizing antibodies targeting quaternary epitope of dengue E protein. Domains I, II, and III are schematically indicated with red, yellow, and blue colors, respectively. Both the top and the side view are displayed one ‘head to tail’ dimer of dengue E protein. Seven of the neutralizing antibodies are shown here schematically that target quaternary epitope in both top and side view of an E dimer. The antibodies which were identified without any ADE effects are shown with red letter code name.

In addition to cross-reactive and serotype-specific antibodies, a novel class of highly neutralizing mAbs has been reported that recognize the viral particle rather than a particular epitope of the envelope protein (106). These antibodies are referred to as quaternary structure-specific because they specifically recognize a certain structural component of viral surfaces (**Figure 5**) (85, 107). For instance, 5J7 is a strongly neutralizing DENV3-specific antibody that detects a cryptic quaternary structure located at an inter-molecular junction of the dimeric E protein. This structural element includes the hinge region residues Gln269 and Asn270 of one envelope molecule A, the domain III lysine residues of positions 310 and 317 of another envelope molecule B, and the domain II tip residues Glu121 and Glu126 of adjacent dimeric molecule B’ (80, 108). Likewise, two of these Fab variants from 5J7 bind tightly to two dimeric unit of envelope proteins made up molecules A, B, A’ and B’ to impede infection by preventing membrane fusion at the post-attachment stage.

Several other reported antibodies, specially 1F4 and 14c10, also bind to the quaternary structure epitope of DENV1, determined by their binding ability only with the whole virus particle rather than with recombinant E protein. Both of these antibodies mainly bind with the domain I and the inter-domain hinge region between domain I and II (**Table 1**). Although 1F4 exclusively binds to a single monomeric envelope protein, it is distinctive among quaternary structure-specific antibodies, while antibody 14c10 binds to two separate E proteins. Protein crystallization studies reveal that both of the antibodies interact with the *β* strand residues of F_0_ and G_0_ and the loop F_0_G_0_ of domain I (**Figure 5**). Since DENV4 specific 5H2 antibody shares binding site mostly with DENV1 specific mAbs 1F4 and 14c10, except the hinge region between domain I and II; hence it might be a critical site for serotype specificity (80). Further research demonstrates that 1F4 is very sensitive to the domain I and II hinge angle and only attaches to the viral particle, not the isolated envelope protein (81).

Moreover, several human mAbs have been identified, which bind with some anti-parallel dimeric epitope or a highly ordered DENV surface quaternary structure. For example, 2D22, a highly neutralizing DENV2 specific mAb, interacts with eight adjacent residues, including R323 in the domain III of one monomer, and fusion and bc loop in the domain II of the adjacent anti-parallel monomers. Although the contact residues of 2D22 in domain III are not conserved among all DENV serotypes, but the residues in domain II are highly conserved between DENV2 and DENV4 (107). These bilateral characteristics of the quaternary epitopes so far determine both the serotype specificity and the neutralization potency (82). Similarly, another DENV2 specific neutralizing mAb 1L12 binds with highly ordered quaternary structure epitopes on the DENV surface E homodimers. They may detect adjacent or overlapped epitopes on the viral surface because 2D22 interfere 1L12 from binding to DENV2 (59).

In contrast, four E-dimer specific mAbs (A11, B7, C10, and C8) have been identified, which are not serotype-specific, however, can potentially neutralize all the four DENV serotypes (**Figure 5**). The contact sites of all these antibodies are aligned in a valley bordered by *β* strand on domain II and *β* strand E_0_F_0_ of domain I. In domain II, both of the E-dimer engages in binding with the same residues of the *β* strand (67-74 with Asn67 glycan), fusion loop, immediate upstream residues from position 97 to 106), and ij loop residues from 246 to 249 position, **Figure 3**). In domain I, the target residues within two E dimers are different; EDE2 interacts with residue N150 in the loop spanning from 148 to159 with Asn150 glycan chain, whereas EDE1 engages with domain I and III to displace Asn150 loop that facilitate antibody binding. Notably, the Asn67 and Asn153 glycans of the E protein, which are conserved throughout the four DENV serotypes, interact extensively with these antibodies. Probably, due to this conserved interaction, the E-dimer-specific mAbs are cross-neutralizing (83). In addition, two broadly neutralizing antibodies, J8 and J9, are also recently reported to neutralize all the four serotypes in the pico-molar range. These antibodies majorly interact with some novel residues in domain I that are distinct from previously described epitopes. Since both of these antibodies predominantly attach to viral particles but not to soluble E protein, the specific location of the proper binding site remains unknown. Therefore, it suggests that the epitope might be quaternary in structure but it needs to be centered in the E monomer (84).

## 4 Pathogenesis and antibody-dependent enhancement of DENV

**Figure 6:**
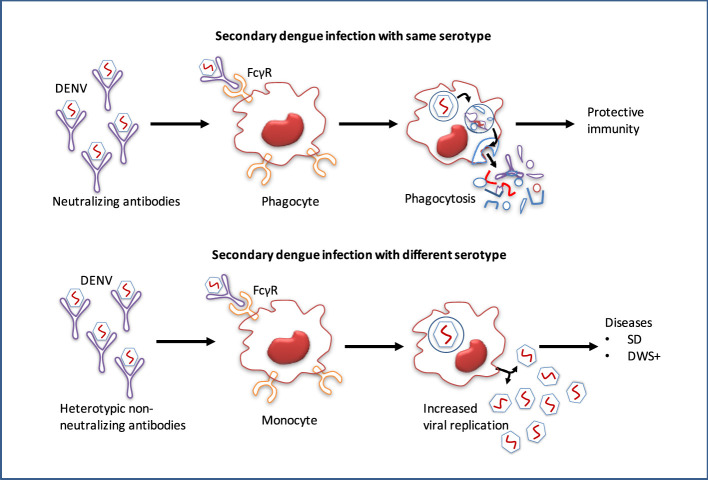
Pathogenesis and antibody-dependent dengue virus enhancing model. While antibodies of primary dengue virus infection bind to an infectious DENV particle during a subsequent infection with the homologous serotype, protective immunity is conferred. However, secondary dengue virus infection with alternative serotypes unable to completely neutralize the virus particle. Instead, the Ab–virus complex interacts to the Fc receptors (FcγR) on circulating monocytes, making it simpler for the virus to infect them, leads to increased viral replication and a higher risk of dengue with warning signs (DWS+)/severe dengue (SD).

After minor clinical issues, the majority of primary dengue patients normally recover, however, the infection confers lifetime protection against that serotype (7). Conversely, re-infection with an alternative serotype not only provides low protection but also leads to more severe diseases (8). Infection with DENV or any other flavivirus, especially ZIKV induces widely cross-reactive but weak or non-neutralizing antibodies, and these antibodies remain detectable for a long period of time (109). During secondary infection, antibodies produced at the initial flavivirus infection may cross-react with alternative serotypes, although they are not entirely neutralizing. These cross-reactive antibodies can thereby increase the uptake of heterologous viruses into the host immune cells, leading to an over-reactive immune response that results in plasma leakage and potentially fatal hypovolemic shock (110). The non-neutralizing antibodies produced during the initial infection may cross-react with the virus of subsequent infections. This virus-IgG immune complexes may then be internalized into myeloid cells through FcγR binding, and increased viral replication leading to the ADE (**Figure 6**) (10).

The ADE hypothesis states that the antibodies produced during primary infection are insufficient to prevent subsequent infections with alternative serotypes that only have 30–35 percent sequence variation. Instead, this low level of cross-reactive non-neutralizing antibodies can exacerbate the condition by accelerating FcγR-mediated endocytosis into the monocyte cells (111). The Fc receptor-mediated pathway of ADE was also found to suppress the expression of anti-inflammatory cytokines, including IL-12, IFN-γ, and innate anti-DENV mediators, nitric oxide radicals, allowing unrestricted DENV replication in the monocyte cell. Although the expression of the anti-inflammatory cytokines IL-6 and IL-10 was facilitated by this route, it is not clear yet how these cytokines contribute to DENV replication (112).

A second DENV infection in children increases the risk of SD 10 times more than a first infection (113). Infants under one-year old who have DENV antibodies from their moms have a greater chance of developing SD than infants born to mothers who have never had the virus (114). Since ADE is an important cause of SD, it poses a fundamental challenge in the development of vaccines. The C-prM-E protein, which is the major component of the DENV vaccine, and the presence of prM antibodies in the host after immunization may cause ADE upon secondary infection with atypical dengue (115).

In addition, the memory T cells, either type-specific or cross-reactive, are produced at initial infection. Contrarily, in repeated DENV infections, viral antigens are produced on the surfaces of infected cells, activating cross-reactive memory T cells, and causing the release of TNF-α and other pro-inflammatory cytokines that cause plasma leakage in the endothelium (116). One of the main characteristics of dengue with warning signs (DWS+) is plasma leakage, which is thought to be caused by vascular endothelial damage brought on by pro-inflammatory cytokines (117). While the precise cause of SD infection is uncertain, it is believed that re-infection with an alternate DENV serotype or even new homologous variants are major alarming factors for SD; this is most likely owing to the ADE (118).

## 5 Scopes of antibody engineering against DENV

The American Food and Drug Administration (FDA) has licensed more than 79 mAbs for curing a wide variety of illnesses, ranging from cancer to chronic inflammatory syndrome and infection to other neurological disorders (119). Many mAbs have shown promising results against several infectious diseases as well as cancer in preclinical evaluation (20, 120). Currently, more than 570 mAbs are under clinical development (121). Out of these, 38 mAbs are under active clinical process against several infectious diseases, including HIV, human respiratory syncytial virus (RSV), SARS, coronavirus, rabies virus, WNV, anthrax, influenza, hepatitis and Ebola (120). Even so, during the Covid-19 pandemic, the FDA has approved convalescent plasma treatment for seriously sick patients, which primarily entails possible antibody collection from recovered individuals (122).

Although a variety of dengue mAbs that are either cross-neutralizing or serotype neutralizing have been proven, decreasing ADE is still a significant difficulty (123). Recently, protein-engineering technologies have been exploited to eliminate ADE and to improve therapeutic efficacy (124). Fc modifications have been commonly used to reduce interaction between Fc and FcγR, which is the most leading cause of ADE. Currently, several engineered antibody variants such as scFv, Fab, modified Fc conjugates, and bispecific constructs are under investigation against several infectious diseases. Substitution or deletion mutations in Fc regions or replacement with IgG4 Fc have been shown to reduce ADE (109, 125). A single point mutation at position 297 (Asn297Gln) in a human mAb, B3B9, has resulted to cross-neutralize all variants of DENV without ADE enhancing effect (70). Similar to this, it is discovered that a widely neutralizing human IgG1 mAb, D23-1G7C2-IgG1 with a mutant Fc (Asn297Ala), impairs the binding to FcγR, which consistently has lower ADE than wild-type (24). Double substitutions from leucine to alanine (LALA) at positions 234 and 235 in the IgG Fc domain have also been demonstrated to abolish contact with FcγR (50). Current research shows that the MZ4 LALA version of the ZIKV antibody has strong neutralizing power against both DENV-2 and ZIKV without encouraging ADE (81).

In contrast, there are two bispecific antibody platforms (DART and DVD-Ig), which have been utilized to prepare therapeutic antibodies against DENV (126). DART (dual-affinity retargeting molecule) is made up of two modified antibody fragments that are connected by short peptide linkers, and each of the chains has a cysteine residue at the C terminus to facilitate the creation of inter-Fv bonds (127). In comparison, DVD-Ig (dual variable domain immunoglobulin) consists of typical IgG with an additional VH/VL domain of another specificity linked by peptide linkers with compatible heavy or light chains. For example, DII-FL specific antibody E60 and A strand of domain III specific antibody 4E11 have been used to develop a bispecific and tetravalent Ig-DART molecule, which retains its *in vitro* neutralizing activity as well as *in vivo* therapeutic activity (84). Along with the resulting construct, an engineered Fc region with the beneficial point mutation (N297Q) could eliminate the ADE (70, 128). Moreover, a novel bispecific DVD-Ig molecule 1A1D-2A10 has been created using two additional sets of well-characterized anti-DENV mAbs, 2A10G6 (2A10) and 1A1D-2 (1A1D). The 2A10 antibody binds to domain II to prevent the virus from fusing with the endosomal membrane, whereas the 1A1D antibody attaches to domain III to limit the viral attachment to the host cell surface (129). Further, an engineered Fc domain with nine residues deletion from the N terminus (position 231-239) abrogates binding with FcγR (109). The resultant design (DVD-1A1D-2A10) is able to neutralize all four DENV serotypes without causing any ADE since it targets both the attachment and fusion phases (129). Therefore, it suggests that these bispecific molecules have great potential against DENV specific antiviral designing.

Finally, some of the Fc modifications are in charge of increasing the serum half-life of mAbs. Fc mutation at His310 and His415 may change the interaction capability to its salvage receptor, FcRn, and prevent lysosomal degradation by redirecting the antibodies for recycle back into the blood stream (130). While mutation at position 250 (T250Q) and 428 (M428L) increases binding affinity with FcRn and enhance the serum half-life by at least two-fold in rhesus monkey without affecting ADCC and CDC (131, 132). Besides, some of the Fc modifications can also alter the binding of ADE inducing complement component C1q. Mutations at positions 326 (K326W) and 333 (E333S) in the epicenter of the C1q binding site are observed at least five-fold improvement in terms of binding and CDC effects, without influencing ADCC (133). Therefore, combining all of these beneficial mutations in the Fc region may improve the effector functions of DENV mAbs and simultaneously may reduce the risk of ADE.

The process of producing antibodies involves the selection of B cells, their proliferation, and differentiation into plasma cells. The process of affinity maturation, takes place primarily in antigen-selected germinal center B cells through somatic hypermutation (SHM) (134). A study determined immunoglobulin heavy chain variable frequency usage and SHM levels using high-throughput sequencing of peripheral blood IgG antibody repertoires from DENV patients. The study reported overall low proportion of somatic hypermutated antibody genes during the acute phase plasmablasts, particularly in secondary infections and those cases with more severe disease (135). So, lower SHM will result in antibodies with suboptimal avidity and affinity towards the epitopes of the secondary-infecting virus thus favouring ADE. The generation of skewed antibody response towards the memory of primary infecting serotype and lower affinity response for secondary infecting serotype due to lower SHM is attributed to original antigenic sin (14).

## 6 Conclusion and future directions

The antibodies targeting serotype-specific epitopes are mostly neutralizing, however, none of them are completely free from the risk of ADE. A few of the cross-reactive antibodies such as Sign3c, 2H12, and VIS513, either targeting the fusion loop of domain II or A strand of domain III, are highly neutralizing, and characterized with minor or no enhancing activities. Some of the quaternary structure specific antibodies including m366.3 and 3E31 are also found cross-neutralizing without any ADE effects. The engineered version of fusion loop specific B3B9 antibody (N293Q-B3B9 rIgG) is also found to be protective against all of the four serotypes without viral enhancing activities. The epitope-paratope binding analyses of this antibody could provide valuable insights which would be helpful in designing of the antigen for the effective vaccine development. Moreover, this information along with the sequence alignment suggest that the fusion loop of domain II and A strand of domain III are highly conserved and generate cross-reactive antibodies. The antibodies generated against these two epitopes are highly neutralizing and mostly they have potential to escape viral enhancing activities.

The detailed description of the most potent and cross-reactive antibodies along with the information of ADE enhancing or reducing epitopes may be beneficial for the discovery of next-generation antibody therapeutics. On the other hand, with a detailed understanding of the binding sites, the potential immune responses, and associated risk analysis may be useful for designing subunit vaccines, possibly in the way of heterologous prime-boosting strategies that can be administrated by following the sequential responses of natural infections. In addition, antibody engineering, especially Fc modifications are currently being utilized to improve therapeutic efficacy either by reducing the risk of ADE or by increasing half-life and effector functions. Although cross-reactive antibodies may increase viremia at sub-neutralizing concentrations and serotype-specific antibodies prevent viral fusion with lower risk of ADE, these dual phenomena can be utilized for therapeutic application by controlling its applications as well as subsequent doses. Finally, antibody cocktail targeting the potential epitopes may also be useful to reduce the chances of neutralizing escape variant, which may be protective against diverse DENV strains regardless of ADE.

## Author contributions

AS and RG contributed to conception and design of the study. AS wrote the first draft of the manuscript. ND contributed by writing a part of the manuscript. RG, AS, and ND revised the manuscript. All authors contributed to the article and approved the submitted version.
